# Monotherapy With Immune Checkpoint Blockade Improves Survival Outcomes in KRAS-Mutant but Not KRAS Wild-Type Metastatic Lung Adenocarcinoma: Validation From an Extended Swedish Cohort

**DOI:** 10.1016/j.jtocrr.2025.100880

**Published:** 2025-07-17

**Authors:** Ella A. Eklund, Sama I. Sayin, Jonas Smith Jonsson, Hannes van Renswoude, Jan Nyman, Andreas Hallqvist, Clotilde Wiel, Volkan I. Sayin

**Affiliations:** a Department of Surgery, Institute of Clinical Sciences, Sahlgrenska Center for Cancer Research, University of Gothenburg, Gothenburg, Sweden; b Wallenberg Centre for Molecular and Translational Medicine, University of Gothenburg, Gothenburg, Sweden; c Department of Oncology, Sahlgrenska University Hospital, Gothenburg, Sweden; d Department of Oncology, Institute of Clinical Sciences, University of Gothenburg, Gothenburg, Sweden

**Keywords:** Lung cancer, KRAS, Chemotherapy, Immunotherapy, Biomarker

## Abstract

**Introduction:**

Immune checkpoint blockade (ICB) is a standard first-line treatment for stage IV NSCLC without actionable oncogenic alterations. *KRAS* mutations, prevalent in 30% to 40% lung adenocarcinomas (LUAD) in Western populations, currently lack targeted first-line therapies. This study aimed to assess the predictive value of *KRAS* mutations for clinical outcomes after distinct ICB regimens, validating our previous findings in a larger cohort with extended follow-up.

**Methods:**

We conducted a retrospective multicenter study including consecutive stage IV LUAD patients (n = 424) treated with either ICB or platinum-doublet chemotherapy between 2016 and 2021 in Western Sweden. Patient demographics, tumor characteristics, treatment details, and survival outcomes were retrospectively collected from patient charts and the Swedish National Lung Cancer Registry. *KRAS* mutational status was assessed by next-generation sequencing. Primary end points included overall survival (OS) and progression-free survival (PFS), analyzed using Kaplan-Meier curves and multivariate Cox regression.

**Results:**

Among 424 patients diagnosed with metastatic LUAD, 40% harbored *KRAS* mutations (*KRAS*^MUT^). *KRAS*^MUT^ patients exhibited significant improvement in OS (16 versus 8 mo, *p* < 0.001) and PFS (8 mo versus 5 mo, *p* < 0.001) with ICB monotherapy. In contrast, *KRAS* wild-type (*KRAS*^WT^) patients derived no survival advantage from ICB monotherapy (OS, 8 mo versus 8 mo, *p* = 0.648; PFS 4 mo versus 5 mo, *p* = 0.871) although they did so with chemoimmunotherapy (OS, 15 mo versus 8 mo, *p* = 0.032; PFS, 6 mo vs 5 mo, *p* = 0.033). On multivariate analysis, monotherapy was confirmed as an independent factor improving outcomes in KRAS-mutated patients (hazard ratio [HR] 0.533, 95% confidence interval 0.311-0.912, *p* = 0.018). Finally, we identified *KRAS*^G12C^ (OS: 13.7 mo versus 10.5 mo, *p* = 0.0046, PFS: 7.7 mo versus 6.2 mo, *p* = 0.002) and *KRAS*^G12V^ (OS: 24.2 mo versus 7.2 mo, *p* = 0.0204; PFS: 13.7 mo versus 4.5 mo, *p* = 0.063) but not *KRAS*^G12D^ (OS, 5.8 mo versus 6.2 mo, *p* = 0.777; PFS, 4.6 mo versus 3.2 mo, *p* = 0.694) as distinctly and independently predictive of improved survival after receiving ICB-containing treatment.

**Conclusions:**

*KRAS* mutations predict substantial and sustained clinical benefit from first-line ICB monotherapy in metastatic LUAD, whereas *KRAS* wild-type patients do not. *KRAS*^G12C^ and *KRAS*^G12V^ mutations confer improved survival, whereas *KRAS*^G12D^ does not. Integrating *KRAS* mutation status into clinical practice could guide personalized treatment strategies, optimizing immunotherapy outcomes in stage IV LUAD.

## Introduction

NSCLC remains the leading cause of cancer-related mortality worldwide, with lung adenocarcinoma (LUAD) representing the predominant histologic subtype.^1^ Recent advancements in molecular profiling and immune checkpoint blockade (ICB) have considerably transformed the therapeutic landscape for patients diagnosed with advanced-stage NSCLC. Currently, ICB—administered either as monotherapy or in combination with chemotherapy or another ICB—constitutes the mainstay first-line treatment option for patients with stage IV NSCLC lacking targetable oncogenic alterations.^2^ However, only a subset of this patient population responds to ICB treatment,^3^ and it is crucial to study long-term follow-up outcomes to identify factors predicting treatment response.

Molecular profiling has become a cornerstone of lung cancer diagnostics, guiding therapeutic strategies by identifying actionable genetic alterations. According to current European Society for Medical Oncology guidelines, patients harboring actionable mutations, such as *EGFR* or *ALK* rearrangements, should initially receive targeted tyrosine kinase inhibitors.^4^ However, these actionable driver alterations are present only in a minority of patients with NSCLC, whereas the majority harbor nonactionable genetic alterations. For the patient subgroup without actionable mutations, guidelines recommend first-line ICB monotherapy for patients with high programmed death-ligand 1 (PD-L1) tumor proportion scores (TPS) (≥50%) or chemoimmunotherapy regimens irrespective of PD-L1 expression.^2^ Still, response to ICB varies substantially between patients, and currently used predictive biomarkers, such as PD-L1 expression and tumor mutational burden (TMB), remain inconsistent predictors of immunotherapy benefit.

*KRAS* mutations represent the most frequently observed oncogenic mutations in NSCLC, occurring in approximately 30% to 40% cases among Western populations.^5^^,^^6^ The role of *KRAS* mutations, alone or in combination with other risk factors, as prognostic markers for clinical outcomes remains a focus of current research.^7^, ^8^, ^9^ Despite their high prevalence, therapeutic strategies specifically targeting *KRAS*-mutant tumors have only recently begun to emerge, leaving immunotherapy-based regimens as the primary first-line treatment option for these patients.^10^^,^^11^ More importantly, *KRAS* mutations have been found to directly interact with the tumor microenvironment (TME) and affect immunogenicity.^12^, ^13^, ^14^ Nevertheless, the prognostic and predictive significance of *KRAS* mutations in the context of real-world outcomes after different ICB-containing treatment regimens remains unclear, and several ongoing trials are evaluating the impact of *KRAS* mutations on response to treatment with ICB.^15^^,^^16^ In our previous study, we reported an increased benefit from ICB-containing treatment among patients with *KRAS*-mutated LUAD compared with those without such mutations, whereas ICB monotherapy seemed less effective in *KRAS* wild-type patients.^17^

In the current retrospective multicenter study, we extend our previous investigation by assembling an expanded cohort comprising all patients diagnosed with stage IV LUAD who underwent molecular assessment between 2016 and 2021 in Western Sweden. By integrating comprehensive data from the Swedish National Lung Cancer Registry with detailed curation from hospital patient charts, our aim was to assess long-term survival outcomes and elucidate the predictive value of *KRAS* mutational status regarding clinical response to ICB-containing treatments. Furthermore, we sought to validate our earlier observations on the role of *KRAS* mutations in a larger patient cohort with extended follow-up, thereby providing robust, real-world clinical evidence to guide treatment decisions and future biomarker-directed therapeutic strategies.

## Materials and Methods

### Patient Population

We conducted a multicenter retrospective study including all consecutive patients with NSCLC LUAD diagnosed with stage IV NSCLC and having molecular assessment performed between 2016 and 2021 in the Region of Västra Götaland (region of West Sweden), Sweden (N= 912). The patient receiving any ICB-containing treatment or platinum-doublet (PD) chemotherapy was selected for the study (n = 432). For inclusion, one treatment cycle was deemed sufficient. Approval from the Swedish Ethical Review Authority (Dnr 2019-04771 and 2021-04987) was obtained before study commencement. All patients with available tissue samples in which an adenocarcinoma component could not be excluded were systematically assessed with next-generation sequencing (NGS) for known genetic drivers (see below) within clinical praxis. Patient demographics (including age, sex, Eastern Cooperative Oncology Group [ECOG] performance status and smoking history), cancer stage, number of metastasis locations, pathologic details (PD-L1 grade, histologic diagnosis, mutation status including *KRAS* mutational status, and subtype), treatment and outcome data were retrospectively collected from patient charts and the Swedish Lung Cancer Registry.

### Mutational Status

Patient selection was done through the pathology regional laboratory information system, in which they were referred for molecular assessment. The selected patients underwent NGS on DNA from formalin-fixed paraffin-embedded blocks or cytologic smears using the Ion AmpliSeq Colon and Lung Cancer Panel version 2 from ThermoFisher Scientific (Waltham, MA) until 2019 and thereafter the Thermo Fisher OncomineTM Focus Assay, assessing hotspot mutations in *EGFR*, *BRAF*, *KRAS,* and *NRAS*. Until June 2017, *ALK*-fusions were assessed with immunohistochemistry, and with fluorescence in situ hybridization if positive or inconclusive immunohistochemistry; *ROS1* was analyzed on request with fluorescence in situ hybridization. Thereafter, *ALK*, *ROS1,* and *RET* fusions were assessed on RNA using the Oncomine Solid Tumor Fusion Panel from ThermoFisher Scientific. The analyses were done as part of the standard diagnostic workup process at the Department of Clinical Pathology at Sahlgrenska University Hospital.

We stratified the patient population in our analyses by the following KRAS mutational status and subtypes: patients with no mutations detected in KRAS (*KRAS*^WT^), mutations in KRAS (*KRAS*^MUT^), and subtypes of G12C (*KRAS*^G12C^), G12V (*KRAS*^G12V^), G12D (*KRAS*^G12D^), and G12A (*KRAS*^G12A^).

### ICB-Containing Treatment

During the time period of this study, the most typically used ICB-containing treatments approved for first-line treatment were monotherapy or chemoimmunotherapy. In addition, anti–CTLA-1 in combination with anti–programmed cell death protein 1 (PD-1) therapy was approved in November 2020, but was rarely used in this cohort. The most common monotherapy is pembrolizumab, a humanized antibody targeting PD-1, for patients with PD-L1–high TPS of greater than or equal to 50%. In addition, atezolizumab, a humanized antibody targeting PD-L1, is also approved for monotherapy for patients with PD-L1 TPS greater than or equal to 20%. Chemoimmunotherapy, which is a combination of carboplatin, pemetrexed, and pembrolizumab, is approved for all patients regardless of PD-L1 TPS.

### PD Treatment

PD treatment consists of carboplatin or cisplatin in combination with one more nonplatinum chemotherapy agent such as pemetrexed, vinorelbine, paclitaxel, or gemcitabine.

### Study Objectives

The primary outcomes of this study were overall survival (OS) and progression-free survival (PFS). OS was defined as the time interval between the date of first treatment and the date of death from any cause. PFS was defined as the time interval between the date of first treatment and the date of progression or death, whichever came first. Patients alive or lost to follow-up at data collection were censored at last contact.

### Statistical Analysis

Clinical characteristics were summarized using descriptive statistics and evaluated with univariate analysis. Kaplan-Meier survival curves were generated to assess OS and PFS. Log-rank test was used to assess significant differences in OS and PFS between groups. Multivariable Cox regression analysis was conducted to compensate for potential confounders. The median follow-up time was calculated using the reverse Kaplan-Meier method. Statistical significance was set at *p* less than 0.05, and no adjustments were made for multiple comparisons. Data analysis was conducted using IBM Statistical Package for the Social Sciences version 27 (IBM SPSS Statistics, IBM Corp., Armonk, NY) and GraphPad Prism version 9 (GraphPad Software, Boston, MA).

## Results

### Patients and Tumor Characteristics

**Figure 1:**
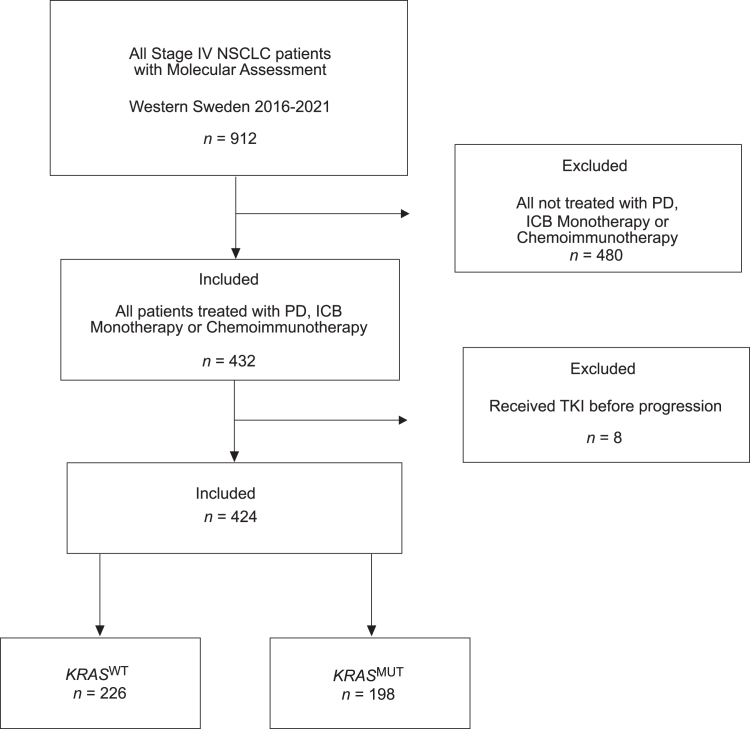
Flowchart illustrating patient selection for the study. ICB, immune checkpoint blockade; PD, platinum-doublet treatment; TKI, tyrosine kinase inhibitors.

A total of 432 patients with LUAD received ICB-containing treatment or PD-chemotherapy in a first-line treatment setting. Eight patients were switched to tyrosine kinase inhibitor treatment before progression and, therefore, were excluded. A total of 424 patients were included in the final study cohort (Fig. 1). Among the included patients, more than 40% harbored a *KRAS* mutation, the majority were female (57%), the median age was 70 years, and most were current or former smokers (88%) (Table 1). There were 68% of patients who had a good performance status with ECOG 0 to 1 at diagnosis (Table 1). The median follow-up time was 67 months. When comparing the baseline characteristics of *KRAS*^WT^ with *KRAS*^MUT^ patients, there were more females, a higher proportion of current and former smokers, and a slightly higher frequency of patients with ECOG 0 in the *KRAS*^MUT^ population. We also observed a higher frequency of patients receiving ICB-containing treatment (30% versus 40%) and a larger proportion with PD-L1–high in the *KRAS*^MUT^ population (Table 1).

**Table 1:** Patient characteristics of the study population. LUAD, lung adenocarcinomas; ECOG, Eastern Cooperative Oncology Group; PD, platinum-doublet treatment; ICB, immune checkpoint blockade; PD-L1, programmed death-ligand 1

All Stage IV LUAD	Total	*KRAS*^MUT^	*KRAS*^WT^
	n (%)	n (%)	n (%)
Total	424 (100)	198 (46.7)	226 (53.3)
Age in years, median (range)	70 (31-87)	71 (46-87)	69 (31-85)
***Sex***			
Male	181 (42.7)	75 (37.9)	106 (46.9)
Female	243 (57.3)	123 (62.1)	120 (53.1)
***Smoking history***			
Current smoker	150 (35.4)	66 (33.3)	84 (37.2)
Former smoker	223 (52.6)	122 (61.6)	101 (44.7)
Never smoker	51 (10.0)	10 (5.1)	41 (18.1)
***Perfomance status***			
ECOG 0	65 (15.3)	35 (17.7)	30 (13.3)
ECOG 1	224 (52.8)	104 (52.5)	120 (53.1)
ECOG 2	107 (25.2)	51 (25.8)	56 (24.8)
ECOG 3	15 (3.5)	4 (2.0)	11 (4.9)
Missing	13 (3.1)	4 (2.0)	9 (4.0)
***Histology***			
Adenocarcinoma	424 (100)	198 (100)	226 (100)
**ICB containing treatment**			
Yes	149 (35.1)	79 (39.9)	70 (31.0)
No	275 (64.9)	119 (60.1)	156 (69.0)
**Treatment**			
Platinum doublet (PD) chemotherapy	275 (64.9)	119 (60.1)	156 (69.0)
ICB monotherapy	75 (17.7)	44 (22.2)	31 (13.7)
Chemoimmunotherapy	74 (17.5)	35 (17.7)	39 (17.3)
***Mutation status***			
None known	186 (43.9)		185 (81.9)
KRAS	198 (46.7)	198 (100)	
EGFR	15 (3.5)		15 (6.6)
BRAF	19 (4.5)	4 (2.0)	15 (6.6)
ALK	2 (0.5)		2 (0.9)
ROS1	6 (1.4)		6 (2.7)
RET	3 (0.7)		3 (1.3)
PIK3CA	3 (0.7)		3 (1.3)
MET	3 (0.7)		3 (1.3)
HRAS Q61R	1 (0.2)		1 (0.4)
***KRAS submutation***			
G12A		12 (6.1)	
G12C		92 (46.5)	
G12D		21 (10.6)	
G12V		33 (16.7)	
G13C		9 (4.5)	
Q61L		9 (4.5)	
Others		22 (11.1)	
***PD-L1 grade (%)***			
*0*	169 (39.9)	72 (36.4)	97 (42.9)
≥ 1	82 (19.3)	37 (18.7)	45 (19.9)
≥ 20	46 (10.8)	15 (7.6)	31 (13.7)
≥ 50	127 (30.0)	74 (37.4)	53 (23.5)
***At last follow up***			
Alive	46 (10.8)	24 (12.1)	22 (9.7)
Deceased	378 (89.2)	174 (87.9)	204 (90.3)
**Survival**			
*Median survival (months)*	9.8	9.8	9.5
***No. of metastatic locations at diagnosis***			
1	277 (65.3)	133 (67.2)	144 (63.7)
2	101 (23.8)	46 (23.2)	55 (24.3)
3	32 (7.5)	15 (7.6)	17 (7.5)
>3	14 (3.2)	4 (2.0)	10 (4.4)

### ICB-Containing Treatment Improves Survival Outcomes in Stage IV LUAD

We initially assessed the efficacy of first-line ICB-containing treatment across the entire study cohort *(n* = 424), and there was a significantly improved OS with a median of 15 months versus 8 months (*p* < 0.001) (Supplementary Fig. 1*A*). In addition, PFS was also significantly longer, 7 versus 5 months (*p* < 0.001) (Supplementary Fig. 1*B*). The results were further controlled for confounders with multivariate Cox regression. We found that ICB-containing treatment was an independent factor for better OS (HR 0.656, 95% confidence interval [CI] 0.511–0.843, *p* = 0.006) and PFS (HR 0.613, 95% CI: 0.482–0.781, *p* < 0.001) (Supplementary Fig. 1*A* and 1*B*).

### Patients With KRAS Mutations Benefit From ICB-Containing Treatment

Next, we proceeded to evaluate the efficacy of first-line ICB-containing treatment separately for *KRAS*^WT^ and *KRAS*^MUT^ patients. For the *KRAS*^MUT^ group, the benefit for OS was 14 months versus 8 months (*p* < 0.001). Similarly, in the *KRAS*^WT^ group, there was an OS benefit with 15 months versus 8 months (*p* = 0.032) (Supplementary Fig. 2*A*). However, when results were further controlled for confounders with multivariate Cox regression, we found that ICB-containing treatment was an independent factor for better OS (HR 0.562, 95% CI: 0.387–0.816, (*p* = 0.002) in the *KRAS*^MUT^ group, whereas it was not an independent factor for better OS in *KRAS*^WT^ (HR 0.716, 95% CI: 0.507–0.1013, *p* = 0.059) (Supplementary Fig. 2*A*).

*KRAS*^MUT^ group exhibited a larger benefit in PFS, 8 months versus 5 months (*p* < 0.001), than in the *KRAS*^WT^ group with 6 months versus 5 months (*p* = 0.033) (Supplementary Fig. 2*B*). When further controlled for confounders with multivariate Cox regression, it confirmed ICB-containing treatment as an independent factor for better PFS both for *KRAS*^MUT^ (HR 0.522, 95% CI: 0.365–0.747, *p* < 0.001) and *KRAS*^WT^ (HR 0.693, 95% CI: 0.496–0.967, *p* = 0.031) (Supplementary Fig. 2*B*).

### Monotherapy With ICB Independently and Significantly Improves Survival Outcomes in KRAS^MUT^ But Not in KRAS^WT^ LUAD

**Figure 2:**
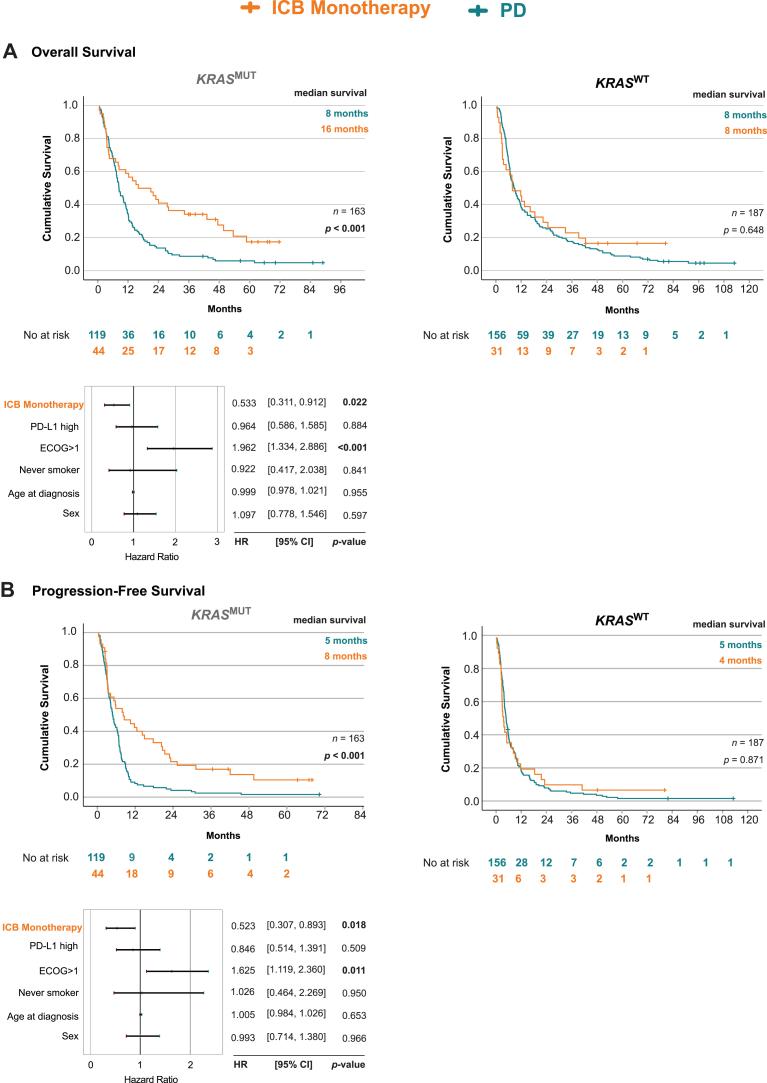
Impact of ICB monotherapy on *(A)* overall survival and *(B)* progression-free survival in LUAD subgroups. Top Panels: Kaplan-Meier estimates survival outcomes between patients who received ICB monotherapy (Yes, orange) or PD alone (No, teal). Bottom Panels: Forest plot of multivariable Cox regression analysis within LUAD subgroups. Left top and bottom panels: *KRAS* mutation (*KRAS*^MUT^), Right top panel: *KRAS*-wild-type (*KRAS*^WT^). ICB, immune checkpoint blockade; LUAD, lung adenocarcinomas; PD, platinum-doublet treatment.

Remarkably, when assessing ICB monotherapy compared with PD treatment, we found that the *KRAS*^MUT^ group clearly benefited with 16 months versus 8 months (*p* < 0.001), whereas the *KRAS*^WT^ group did not benefit, with OS 8 months versus 8 months (*p* = 0.648) (Fig. 2*A*). In multivariate analysis, receiving ICB monotherapy was an independent factor for better OS in the *KRAS*^MUT^ group (HR 0.533, 95% CI: 0.311–0.912, *p* = 0.022) (Fig. 2*A*).

*KRAS*^MUT^ group clearly benefited in PFS from ICB monotherapy, with 8 months versus 5 months (*p* < 0.001) compared with PD (Fig. 2*B*). However, the PFS analysis did not reveal any benefit of ICB monotherapy for the *KRAS*^WT^ group with 4 months versus 5 months (*p* = 0.871) (Fig. 2*B*). In multivariate analysis, receiving ICB monotherapy was an independent factor also for better PFS in the *KRAS*^MUT^ group (HR 0.523, 95% CI: 0.307–0.893, *p* = 0.018) (Fig. 2*B*).

### ICB Treatment in Combination With PD-Chemotherapy Improves Survival Outcomes in KRAS^MUT^ and KRAS^WT^ LUAD

**Figure 3:**
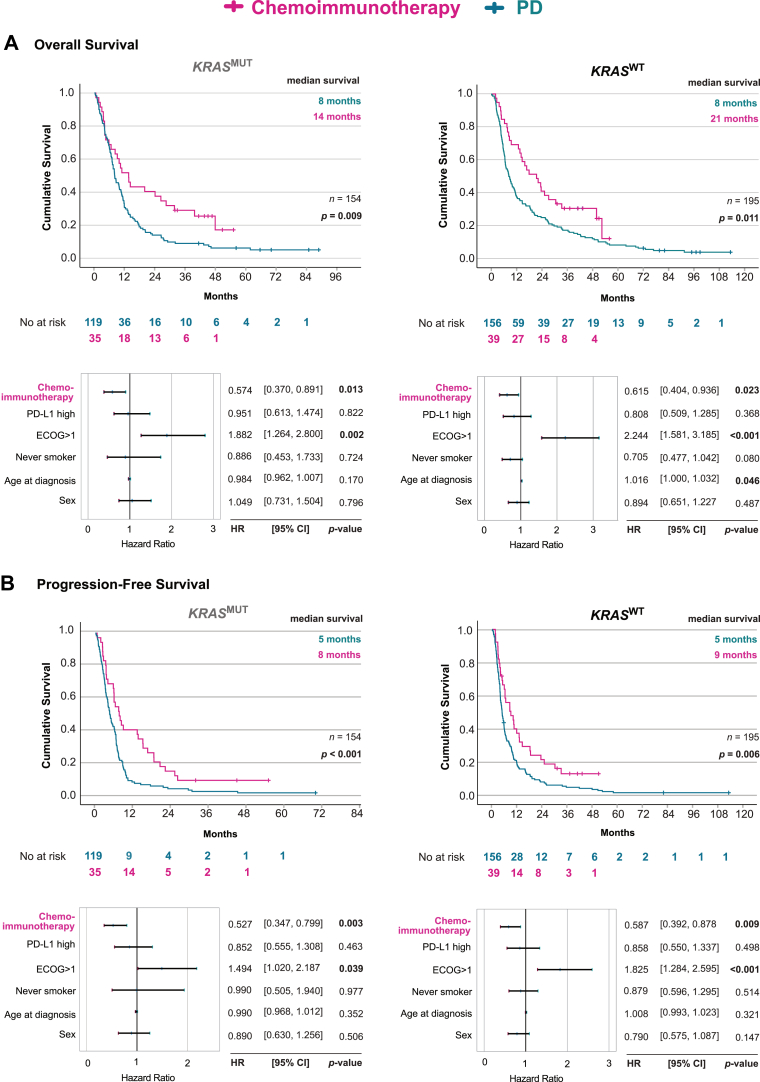
Impact of chemoimmunotherapy on *(A)* overall survival and *(B)* progression-free survival in LUAD subgroups. Top panels: Kaplan-Meier estimates comparing survival outcomes between patients who received chemoimmunotherapy (Yes, magenta) or PD alone (No, teal). Bottom Panels: Forest plot of multivariable Cox regression analysis within LUAD subgroups. Left top and bottom panels: *KRAS* mutation (*KRAS*^MUT^), right top and bottom panels: *KRAS*-wild-type (*KRAS*^WT^). ICB, immune checkpoint blockade; LUAD, lung adenocarcinomas; PD, platinum-doublet treatment.

When we assessed chemoimmunotherapy compared with PD treatment, we found that *KRAS*^MUT^ patients had an improved OS on chemoimmunotherapy compared with PD, 14 months versus 8 months (*p* = 0.009), and *KRAS*^WT^ had an OS of 21 months versus 8 months (*p* = 0.011) (Fig. 3*A*). There was also a benefit in PFS for *KRAS*^MUT^ with 8 months versus 5 months (*p* < 0.001), and 9 months versus 5 months for *KRAS*^WT^ (*p* = 0.006) (Fig. 3*B*). When further controlled for confounders with multivariate Cox regression, chemoimmunotherapy was an independent factor for better OS and PFS for both the *KRAS*^MUT^ and *KRAS*^WT^ groups (Fig. 3*A* and *B*).

### KRAS^G12C^ and KRAS^G12V^ But Not KRAS^G12D^ Mutations Independently Improve Survival Outcomes After ICB-Containing Treatment

**Figure 4:**
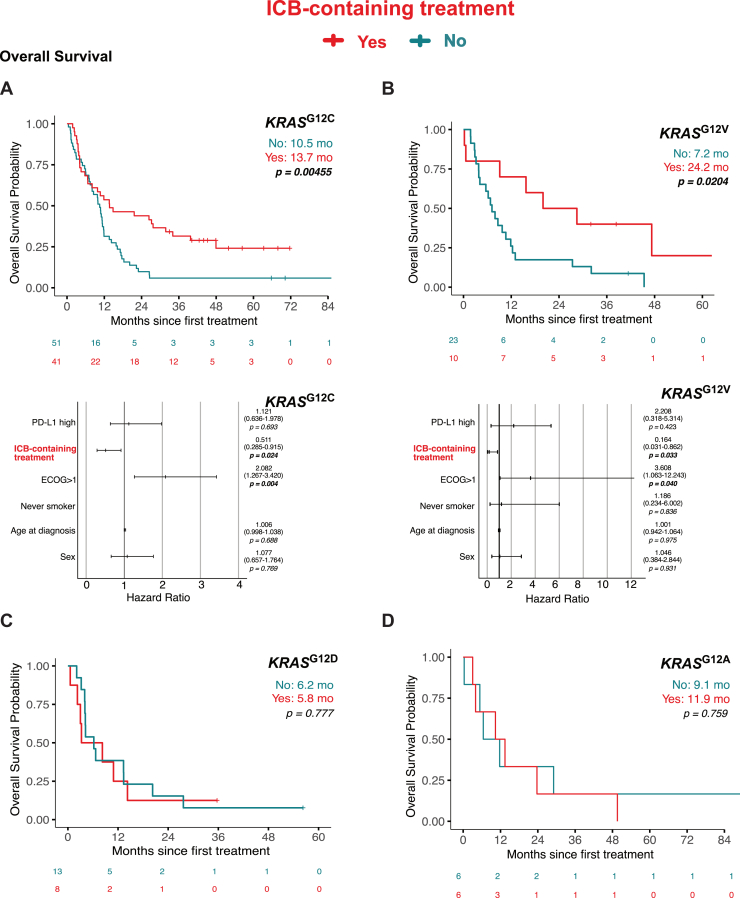
Impact of ICB-containing treatment on overall survival in LUAD KRAS-submutation groups. Top panels *(A-D):* Kaplan-Meier estimates comparing overall survival outcomes between patients who received ICB-containing treatment (Yes, red) or PD alone (No, teal), stratified by KRAS submutations: *KRAS*^G12C^, *KRAS*^G12V^, *KRAS*^G12D^ or *KRAS*^G12A^. Bottom panels *(A-B):* Forest plots of multivariable Cox regression analyses. ICB, immune checkpoint blockade; LUAD, lung adenocarcinomas; PD, platinum-doublet treatment.

**Figure 5:**
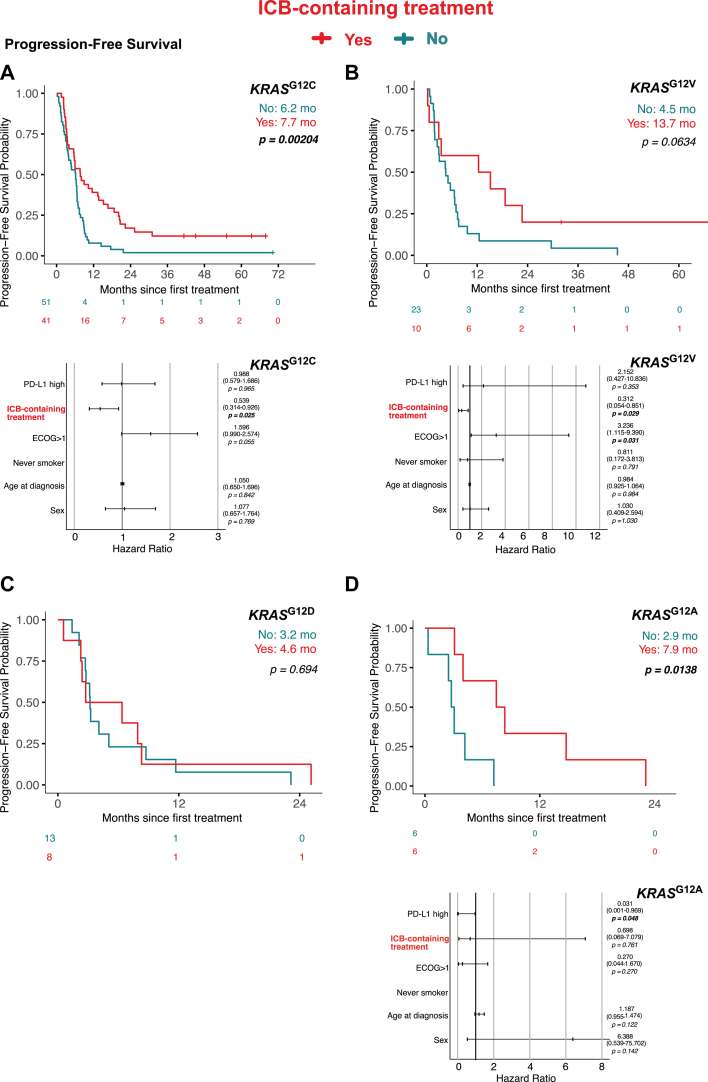
Impact of ICB-containing treatment on progression-free survival in LUAD KRAS-submutations groups. Top Panels *(A-D):* Kaplan-Meier estimates comparing overall survival outcomes between patients who received ICB-containing treatment (Yes, Red) or PD alone (No, Teal), stratified by KRAS submutations: *KRAS*^G12C^, *KRAS*^G12V^, *KRAS*^G12D^, or *KRAS*^G12A^. Bottom panels *(A, B, D):* Forest plots of multivariable Cox regression analyses. ICB, immune checkpoint blockade; LUAD, lung adenocarcinomas; PD, platinum-doublet treatment.

When we stratified survival outcomes by KRAS mutation subtypes, we found that patients with *KRAS*^G12C^ (median OS: 13.7 mo versus 10.5 mo; *p* = 0.0046) and *KRAS*^G12V^ (24.2 mo versus 7.2 mo; *p* = 0.0204) exhibited significantly improved OS with ICB-containing treatment compared with those receiving PD-chemotherapy (Fig. 4*A* and *B*). Similar improvements were observed for PFS in both subgroups (*KRAS*^G12C^ 7.7 mo versus 6.2 mo; *p* = 0.002; *KRAS*^G12V^ 13.7 mo versus 4.5 mo; *p* = 0.063) (Fig. 5*A* and *B*).

Patients with *KRAS*^G12A^ also exhibited significantly improved PFS with ICB (7.9 mo versus 2.9 mo; *p* = 0.0138) (Fig. 5*D*), although this did not translate into a statistically significant OS benefit (11.9 mo versus 9.1 mo; *p* = 0.759) (Fig. 4*D*). In contrast, the *KRAS*^G12D^ subgroup exhibited no improvement in either OS (5.8 mo versus 6.2 mo; *p* = 0.777) or PFS (4.6 mo versus 3.2 mo; *p* = 0.694) with ICB treatment (Figs. 4*C* and 5*C*).

In multivariate Cox regression analyses adjusting for confounders including ECOG performance status, age, sex, PD-L1 status, and smoking history, both *KRAS*^G12C^ and *KRAS*^G12V^ remained independent predictors of improved OS and PFS with ICB treatment. Specifically, *KRAS*^G12C^ was associated with significantly better OS (HR 0.511; 95% CI: 0.285–0.915; *p* = 0.024) (Fig. 4) and PFS (HR 0.539; 95% CI: 0.314–0.926; *p* = 0.025) (Fig. 5). Similarly, *KRAS*^G12V^ exhibited an independent survival benefit in both OS (HR 0.164; 95% CI: 0.031–0.862; *p* = 0.033) and PFS (HR 0.312; 95% CI: 0.054–0.851; *p* = 0.029) (Figs. 4 and 5). Notably, *KRAS*^G12A^ was not an independent predictor of improved PFS after adjusting for confounding variables.

## Discussion

In this extended retrospective, multicenter analysis of all patients consecutively diagnosed with stage IV LUAD in Western Sweden with long-term follow-up, we found that ICB monotherapy significantly and independently improves both OS and PFS in *KRAS*^MUT^ but not *KRAS*^WT^ patients, even though, in comparison, both groups benefit from chemoimmunotherapy. Further, we find that patients with *KRAS*^G12C^ and *KRAS*^G12V^ but not *KRAS*^G12D^ submutations have improved survival with ICB-containing treatment. More importantly, these findings validate and expand our previous work and highlight the crucial predictive role of *KRAS* mutations in response to monotherapy with ICB.

The observed differential response is particularly notable given the historical perception of *KRAS* mutations as a negative prognostic indicator in NSCLC. More recent literature increasingly suggests that *KRAS* mutations may be prognostic for improved outcomes in patients with NSCLC treated with ICB.^18^ A meta-analysis by Zhao et al.^19^ and a study by Li et al.^20^ reported similar OS and PFS improvements in *KRAS*-mutant patients treated with immunotherapy. Sun et al.,^18^ comparing only patients with PD-L1 greater than 50% treated with ICB monotherapy versus chemotherapy, found superior survival in *KRAS*^MUT^ compared with wild-type, and like us, there was no difference in survival between the mutational status when receiving chemoimmunotherapy.

In contrast, Noordhof et al.^21^ found no survival difference by *KRAS* mutation after ICB monotherapy, and Mok et al.^22^ (KEYNOTE-042, Pembrolizumab versus chemotherapy for previously untreated, PD-L1-expressing, locally advanced or metastatic NSCLC) observed clinical benefit from pembrolizumab regardless of KRAS status compared with chemotherapy. However, unlike us, these studies have included mixed NSCLC histologic diagnoses and only patients with PD-L1 greater than 50% (only 30% of the current study population). Given PD-L1 has been found to be an unreliable biomarker of immunotherapy response,^23^ our cohort, not restricted by PD-L1, includes a more representative population for judging the biologic underpinnings of *KRAS*-mutated LUAD response to immunotherapy. In addition, our real-world cohort study provides enhanced robustness because of substantial extended follow-up, reinforcing *KRAS* mutations as positive predictive biomarkers for ICB treatment efficacy.

More importantly, the response within *KRAS*-mutant NSCLC is not universally uniform.^20^^,^^24^ Although *KRAS*^G12C^ is consistently reported as ICB-responsive^19^^,^^25^(a finding our study confirms); our data also reveal a stronger and independent survival benefit for *KRAS*^G12V^ patients (24.2 mo versus 7.2 mo; HR 0.51; *p* = 0.024), which has been inconsistently reported. In a meta-analysis of pooled real-world data, Zhao et al.^19^ found survival outcomes of patients with KRAS^G12V^ comparable to those of patients with KRAS^G12C^ mutation, but significantly longer than those of patients with KRAS^G12D^ mutation. Nevertheless, Sun et al.^18^ noted a moderate or unclear benefit for *KRAS*^G12V^ in direct comparison with other mutational subtypes receiving ICB. In line with this, our findings also confirm that *KRAS*^G12D^ is not associated with improved OS or PFS after ICB-containing treatment. This aligns with reports from Sun et al.,^26^ and Urtecho et al.,^27^ and Peng et al.^25^, all reporting poor ICB response for *KRAS*^G12D^. However, given the relatively small sample size of the submutation groups in our cohort, it is difficult to draw definitive conclusions about *KRAS*^G12A^ or to analyze overall submutational differences in response to monotherapy.

More importantly, oncogenic KRAS signaling mediates autocrine interactions and bidirectional crosstalk with the TME, fostering an inflammatory milieu that may prime tumors for ICB.^28^, ^29^, ^30^ Our findings align closely with Liu et al.^12^ and Torralvo et al.,^31^ who reported enhanced immunogenicity and superior responses to anti–PD-1/PD-L1 therapies in KRAS-mutant cohorts. Specifically, our data confirm previously reported survival benefits with ICB in *KRAS*^G12C^ and particularly *KRAS*^G12V^, which have been found to correlate with elevated PD-L1 expression, higher TMB, and dense CD8+ T-cell infiltration, positioning these as immunologically "hot" phenotypes.^29^ In contrast, *KRAS*^G12D^ tumors exhibited no survival improvement with ICB, also consistent with Sun et al.^26^ and Wang et al.,^32^ who linked this subtype to reduced immunogenicity of the TME through decreased PD-L1 expression, lower TMB, and diminished lymphocyte recruitment, positioning *KRAS*^G12D^ as representing an immune-cold phenotype less amenable to checkpoint blockade.^33^

The superior efficacy of ICB monotherapy in KRAS-mutant versus wild-type LUAD may, in addition, reflect paradoxical immunosuppressive effects of concurrent chemotherapy, potentially blunting the inherent immune activation in the KRAS-driven tumors.^34^ Our findings support prioritizing ICB monotherapy in *KRAS*^G12C^ and *KRAS*^G12V^ subtypes while avoiding combinatorial approaches that might compromise antitumor immunity. However, although our data helps in clarifying subtype-specific response patterns, prospective validation remains critical because of the relatively small subgroup sizes. Finally, evidence also indicates marked heterogeneity in response to ICB related to co-occurring genetic alterations, such as *STK11*, *KEAP1*, *TP53,* and *LRP1B*.^7^^,^^35^^,^^36^ Although our study did not comprehensively evaluate co-mutations, this aspect remains critical for future investigations and biomarker refinement.

Strengths of our study include a large, well-characterized, and regional-level representative patient cohort with comprehensive long-term follow-up data, alongside the utilization of standardized molecular testing practices aligned with routine multidisciplinary clinical care. Limitations include the retrospective nature, potential selection bias, and lack of detailed co-mutation analyses. These limitations indicate important directions for subsequent prospective validation studies.

In conclusion, we find that *KRAS* mutations are strongly predictive of long-term benefit from ICB monotherapy in metastatic LUAD. More importantly, our findings caution against the use of ICB monotherapy in patients without *KRAS* mutations. Specifically, our findings suggest that *KRAS*-mutant patients, particularly those with G12C or G12V mutations, gain substantial and durable survival benefit from ICB alone, whereas *KRAS* wild-type patients seem to derive limited or no benefit from immunotherapy without concurrent chemotherapy. Integrating *KRAS* mutational analysis into clinical decision-making may thus enable more precise, personalized treatment strategies, optimizing immunotherapy outcomes for patients with stage IV LUAD.

## CRediT Authorship Contribution Statement

**Ella A. Eklund**: Conceptualization; Formal analysis; Investigation; Methodology; Supervision; Validation; Visualization; Writing - original draft; and Writing - review & editing

**Sama I. Sayin**: Conceptualization; Formal analysis; Investigation; Methodology; Supervision; Validation; Visualization; Writing - original draft; and Writing - review & editing

**Jonas Smith Jonsson:** Data curation; Formal analysis

**Hannes van Renswoude**: Data curation; Formal analysis

**Jan Nyman:** Conceptualization; Supervision; Writing - review & editing

**Andreas Hallqvist:** Conceptualization; Supervision; Writing - review & editing

**Clotilde Wiel:** Conceptualization; Project administration; Supervision; Funding acquisition; Writing - review & editing

**Volkan I. Sayin:** Conceptualization; Project administration; Supervision; Funding acquisition; Writing - review & editing

## Ethical Statement

Approval from the Swedish Ethical Review Authority (Dnr 2019-04771 and 2021-04987) was obtained before the commencement of the study. No informed consent was required because all data were presented in a deidentified form according to the Swedish Ethical Review Authority.

## Consent for Publication

Not applicable. Patient consent statements were not required because of the retrospective nature of this study, according to the Swedish Ethical Review Authority.

## Declaration of generative AI and AI-assisted Technologies in the Writing Process

During the preparation of this work the authors used ChatGPT4o to proof text and improve language for clarity. After using this tool, the authors reviewed and edited the content as needed and take full responsibility for the content of the publication.

## Disclosure

The authors declare no conflicts of interest.
