# Supplementary material for: Monotherapy With Immune Checkpoint Blockade Improves Survival Outcomes in KRAS-Mutant but Not KRAS Wild-Type Metastatic Lung Adenocarcinoma: Validation From an Extended Swedish Cohort
Source: JTO Clin Res Rep. 2025 Jul 17;6(10):100880. doi: 10.1016/j.jtocrr.2025.100880 (PMC12414821; doi:10.1016/j.jtocrr.2025.100880)
Supplement: Supplementary Figures 1 and 2 [file mmc1.docx]

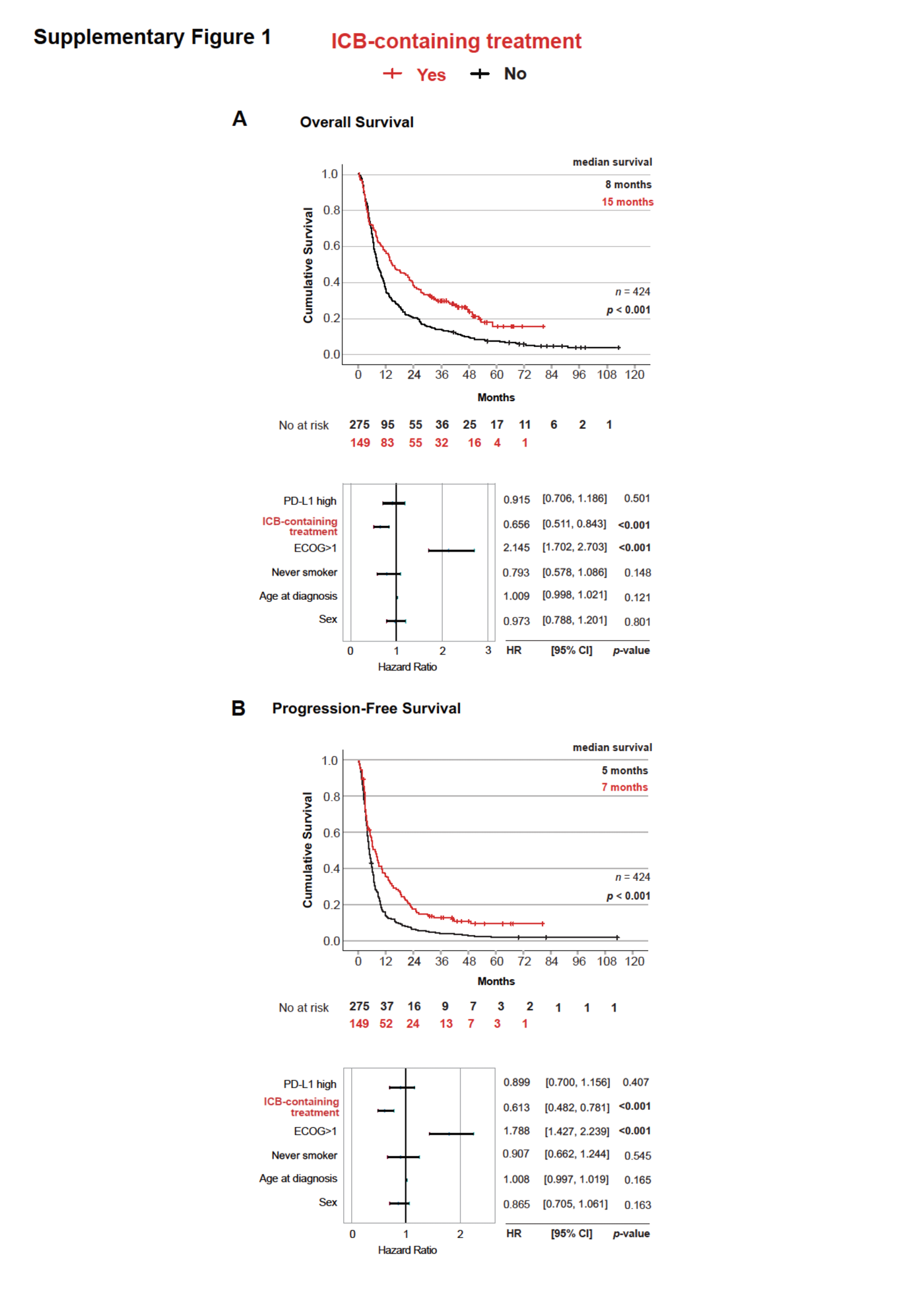
**Supplementary Figure 1**. Impact of ICB-containing treatment on (**A**) overall survival and (**B**) progression-free survival in all LUAD patients. Top Panels: Kaplan-Meier estimates comparing survival outcomes between patients who received ICB-containing treatment (Yes, Red) or PD alone (No, Black). Bottom Panels: Forest plot of multivariable COX regression analysis among all LUAD patients

**Supplementary Figure 2**


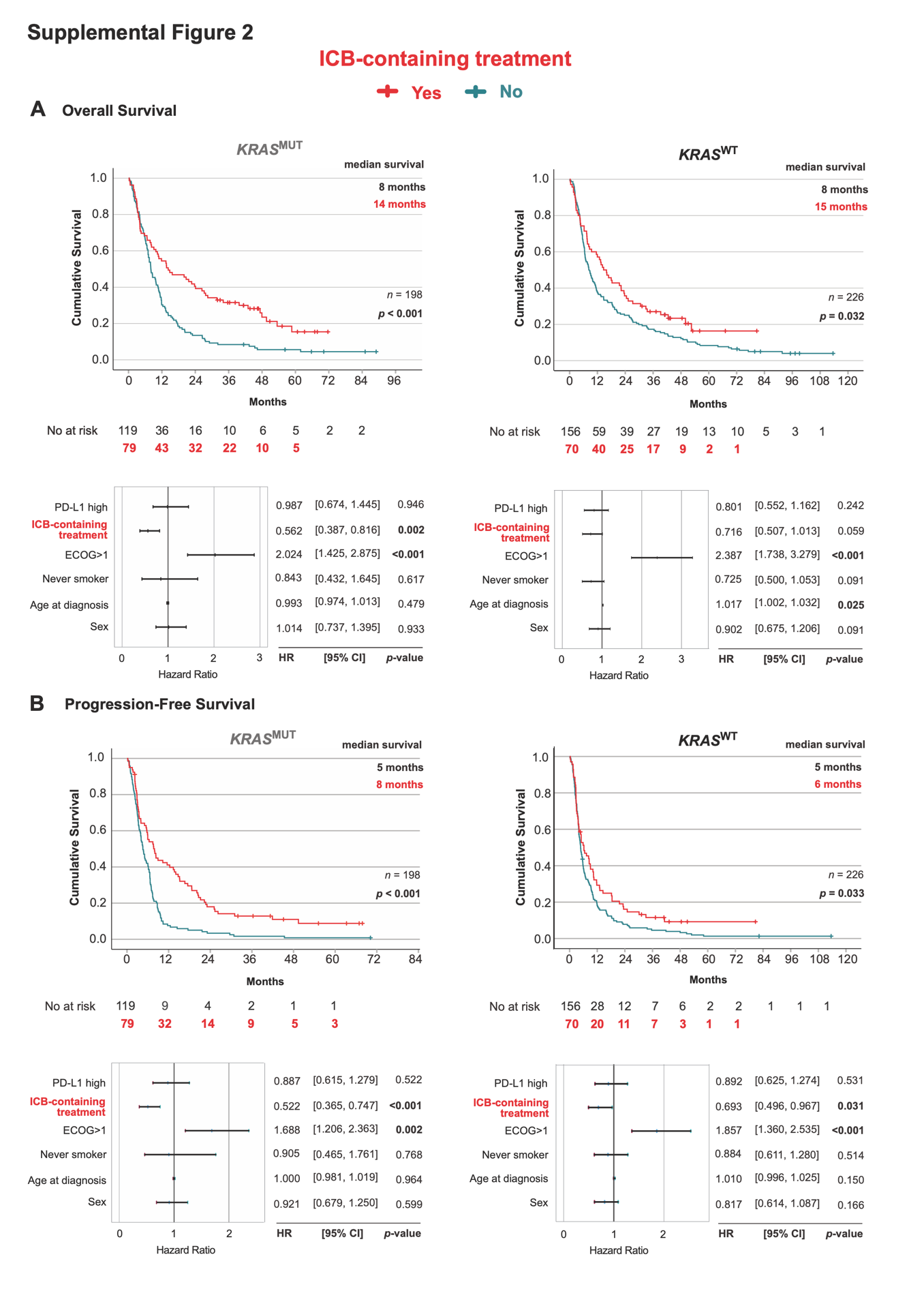


**Supplementary Figure 2**. Impact of ICB-containing treatment on (**A**) overall survival and (**B**) progression-free survival in LUAD subgroups. Top Panels: Kaplan-Meier estimates comparing survival outcomes between patients who received ICB-containing treatment (Yes, Red) or PD alone (No, Teal). Bottom Panels: Forest plot of multivariable COX regression analysis within LUAD subgroups. Left top and bottom panels: KRAS mutation (KRAS^MUT^), Right top and bottom panels: KRAS wild-type (*KRAS*^WT^).
